# Birth and birth-related obstetrical characteristics in southwestern China associated with the current adjustment of family planning policy: a 7-year retrospective study

**DOI:** 10.1038/s41598-020-73039-7

**Published:** 2020-09-29

**Authors:** Xiyao Liu, Dongni Huang, Yu Wang, Yuwen Gao, Miaomiao Chen, Yuxiang Bai, Mengshi Wu, Xin Luo, Hongbo Qi

**Affiliations:** 1 Department of Obstetrics, The First Affiliated Hospital of Chongqing Medical University, No. 1 Youyi Road, Yuzhong District, Chongqing, 400016 China; 2 China-Canada-New Zealand Joint Laboratory of Maternal and Fetal Medicine, Chongqing Medical University, No.1 Yixueyuan Road, Yuzhong District, Chongqing, 400016 China; 3 First Clinical Institute, Chongqing Medical University, No. 1 Yixueyuan Road, Yuzhong District, Chongqing, 400016 China; 4 School of Public Health and Management, Chongqing Medical University, No. 1 Yixueyuan Road, Yuzhong District, Chongqing, 400016 China; 5 Maternal and Child Health Hospital of Hubei Province, No. 745 Wuluo Road, Hongshan District, Wuhan, 430070 Hubei China

**Keywords:** Diseases, Health care, Medical research, Risk factors

## Abstract

In China, the adjustment of the family planning policy was expected to increase the number of births and trigger a change in the demographic and obstetrical background of pregnant women. The policy itself, and corresponding background variations of the pregnant mothers, might have various influences on certain birth-related characteristics. Moreover, the adaption of the medical system to the policy needs to be demonstrated. To address these issues, over 50,000 individual records from January 2012 to December 2018 were collected from a large tertiary care centre of southwest China as a representative. The monthly numbers of deliveries and births showed stabilized patterns after remarkable upward trends. Policy-sensitive women, among whom older age and multiparity were typical features, contributed considerably to the remarkable additional births. Indeed, multivariable logistic regression analysis identified the child policy and these two background characteristics as factors influencing CS (caesarean section) rate and certain pregnancy complications or adverse outcomes. After the implementation of the two-child policy, a care provider was faced with fewer but more difficult cases. Briefly speaking, more individual-based studies on family planning policy and more efforts to improve obstetrical service are needed to better guide clinical practice in the new era.

## Introduction

Launched in 1979, at the beginning of China's economic reforms, China's iconic one-child policy was the continual focus of debates raging over its morality and its controversial social effects. Increasing demographic warnings about the hyper-aging population structure, shrinking workforce and imbalanced sex ratio, which might threaten national economic growth in the twenty-first century, raised the question of whether the one-child policy was ever necessary at all^1,2^. Since November 2013, couples have been allowed to have two children if at least one of the marital partners was an only child (the selective two-child policy)^3^. In October 2015, a more relaxed policy, permitting all couples to have two children (the universal two-child policy), was officially implemented^4^.

Public research, accounting for almost all of the studies on this issue, listed out the policy's consequences on fertility, population, and economic development^5,6^. However, when it comes to the effects on population health and health systems, details have seldom been discussed in depth, especially as related to certain birth-related obstetrical characteristics. A more recent report^7^ described a national, descriptive before-and-after comparative study on the association of China's universal two-child policy with the number of births, the proportions of older (≧ 35 years) and multiparous mothers, and changes in delivery mode. Notably, similar factors, including but not restricted to those mentioned in the article above, are of vital importance to guide and adjust clinical practice. Moreover, analysis of pregnancy outcomes is also clinically applicable and can be better achieved based on individual data.

The effects of policy changes on reproductive health are inevitably speculative. First, with the shifts of these policies and the following increase in the number of births^7,8^, the background characteristics of expectant mothers would change: mothers with advanced age (≧ 35 years)^4^ and with multiparity increased^7^, and variations in the proportions of multiple and of ART (assisted reproductive technology) pregnancies might occur. Second, additional births would call for more maternal and child-health services, which should be planned for as necessary^6^. Third, gaining a second chance at giving birth, families' perception of delivery mode would change, and the frequency of selective abortion might drop due to a weakening of sex preference. Thus, the policy was supposed to rationalize the CS (caesarean section) rate^9^ and the new-born sex ratio^6^. Fourth, given factors stemming from policy changes, such as older age of pregnant women, the reproductive population would bear a higher risk of many complications and adverse pregnancy outcomes^10,11^. Overall, with the aim of seeking more information about the policy's obstetrical effects, some subgroups, known as potential influencing factors, are crucial and need to be analysed thoroughly.

In the context of a series of adjustments of national reproductive strategies, we attempt to describe changes in birth and birth-related obstetrical characteristics, and identify possible reasons for these changes, based on individual data from a large tertiary care centre of southwest China as a representative. Our findings may guide politicians and obstetricians in nearby areas in formulating effective plans to improve maternal and foetal outcomes in the two-child era.

## Results

**Figure 1:**
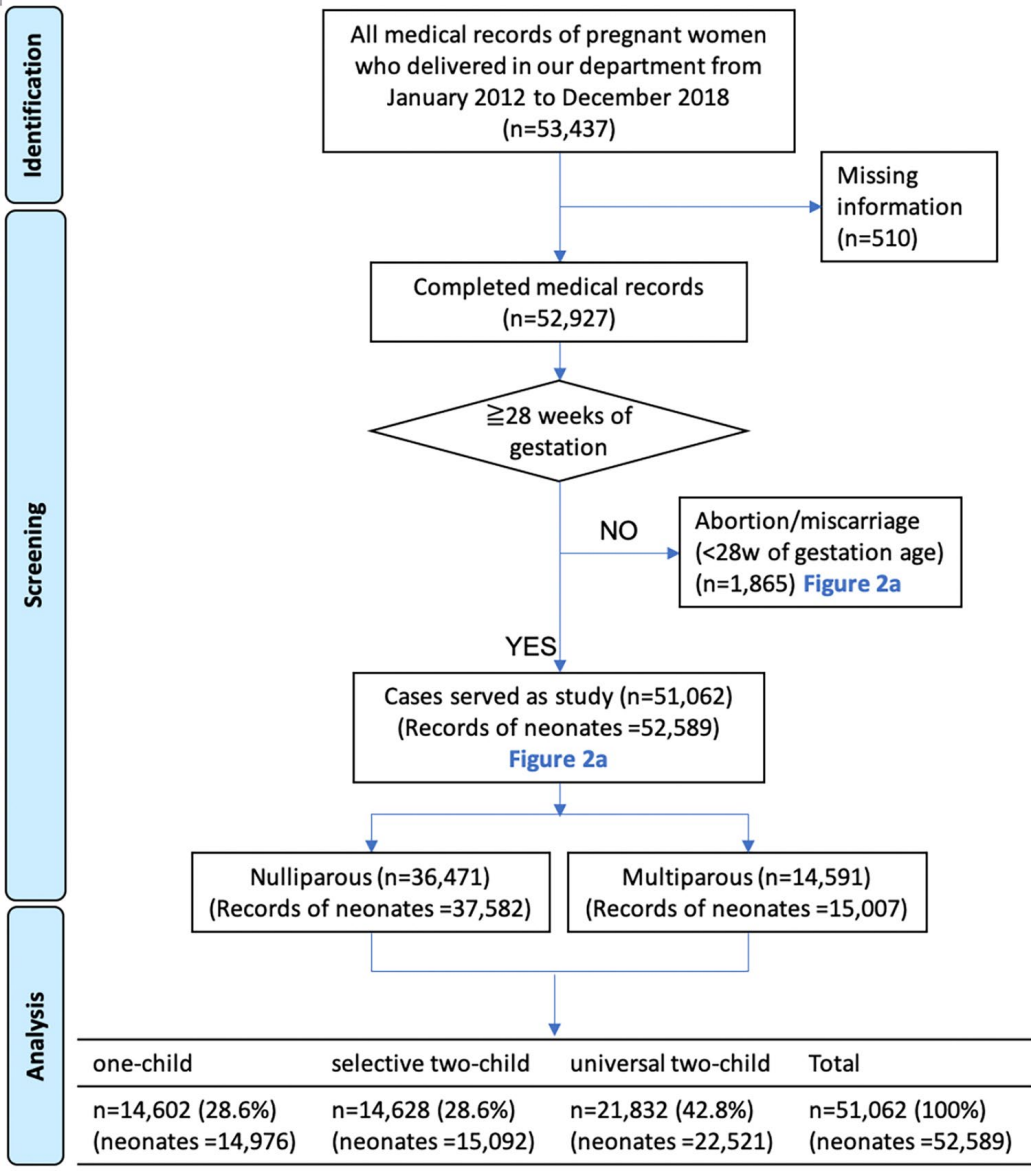
Flowchart of the study.

**Figure 2:**
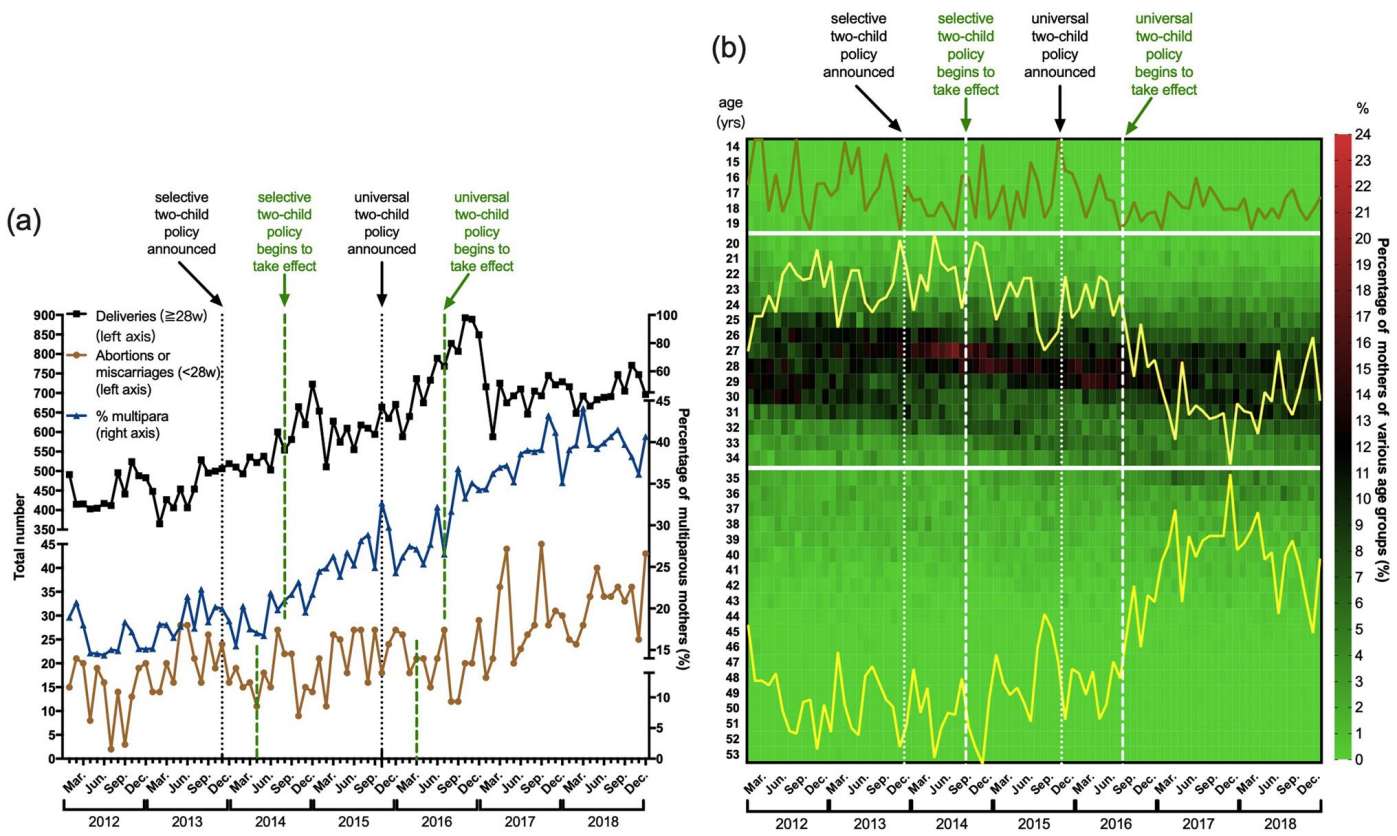
Monthly trends of deliveries and corresponding background of pregnant women (a) The monthly trends of (1) the absolute number of deliveries (gestational age ≧ 28w) (black curve, left axis); (2) the absolute number of abortions or miscarriages (gestational age < 28w) (brown curve, left axis); and (3) the percentage of multiparous mothers (blue curve, right axis). (b) The monthly age constitution of pregnant women. It can be seen as 2 figures. Both the heatmap and the line chart were generated and merged by GraphPad Prism 8 for Windows, Version 8.0.1 (244), URL link: https://www.graphpad.com/. The heatmap shows the detailed distribution of pregnant women of each age: each column represents each month (from year of 2012 to 2018) and each row an age (from 14 to 53 years); the colour, with its colour key at the very right of the figure, indicates the percentage of women of that age among all the delivered mothers in the corresponding month. In the line chart, the X axis (the same as that of the heat map) indicates the time of delivery, while the Y axes (not shown) indicate the percentages of women in the corresponding age groups: (1) lower-age (from 14 to 19 years): a stable curve (ranging from 0 to a peak at 1.21%); (2) right-age (from 20 to 34 years): a declining curve (peak at 91.95% and bottom at 79.89%); (3) advanced-age (35 years or older): an increasing curve (bottom at 7.59% and peak at 19.83%).

The flowchart of the study is shown in Fig. 1. A total of 53,437 records of mothers were identified from the database, with 510 (0.95%) uncompleted records that were excluded from our analysis. The abortion or miscarriage records (1865 records, 3.49%) were selected and excluded by gestational age, the temporal patterns of which are shown in Fig. 2a (brown curve, left axis). Ultimately, 51,062 records (52,589 neonates) in total were considered as our study subjects.

### Trend of the absolute number of deliveries

Of all the cases of pregnancies, 14,602 (28.6%), 14,628 (28.6%) and 21,832 (42.8%) were from the 3 periods of the time series, corresponding to 14,976 (28.5%), 15,092 (28.7%) and 22,521 (42.8%) neonatal records, respectively. Visual inspection of the total deliveries over time (Fig. 2a, black curve, right axis) shows a stable trend in 2012–2013, with some minor fluctuations. After the announcement of these family planning policies, especially the universal two-child policy, a marked increase in deliveries occurred, with the highest peaks in the winters of 2014 and 2016. The delivery trend finally became stable after 2016.

### Change of background characteristics in pregnant women

As shown in Table 1, the percentages of multiple pregnancies and IVF-ET (in vitro fertilization and embryo transfer) were almost consistent among the 3 periods (detailed curves not shown). The most significant differences, both statistically and clinically, were identified in age and parity, the curves of which are presented in Fig. 2b (for age) and 2a (for parity, blue curve to the right axis) in detail. The number of pregnant women of advanced age (≧ 35 and even ≧ 40 years) and of multiparity witnessed a sharp upward trend, and the proportion of "right-age" women (between 20 and 35 years) decreased markedly. This remarkable change took place just after the shift from the selective two-child policy to the universal two-child policy. Moreover, such results indicate that women of advanced age and women having already given birth before were more likely to be the target population of these reproductive policies.

**Table 1:** Background characteristics of pregnant women.

	Periods			P-value
	One-child policy	Selective two-child policy	Universal two-child policy	
**Age groups (years)**^**a**^				< 0.001
< 20	0.5	0.5	0.3	
[20, 35)	89.4	88.7	83.9	
[35, 40)	8.0	8.5	12.9	
≧ 40	2.1	2.3	2.9	
Multiparity^a^	17.8	25.9	37.6	< 0.001
Multiple pregnancy	2.5	3.1	3.1	0.001
IVF-ET	1.8	2.1	2.2	0.021

Data are shown in percentage. Chi-squared test was used.

*IVF-ET* in vitro fertilization and embryo transfer.

^a^Those variables were chosen as subgroup factors and defined as covariates for further analysis.

We finally chose pregnant women of advanced age (≧ 35 years) and multiparous women as two subgroups for analysing additional births. These two factors were considered as covariates when analysing the influence of policy on the birth-related obstetrical characteristics.

### Additional births and care providers' workload

**Figure 3:**
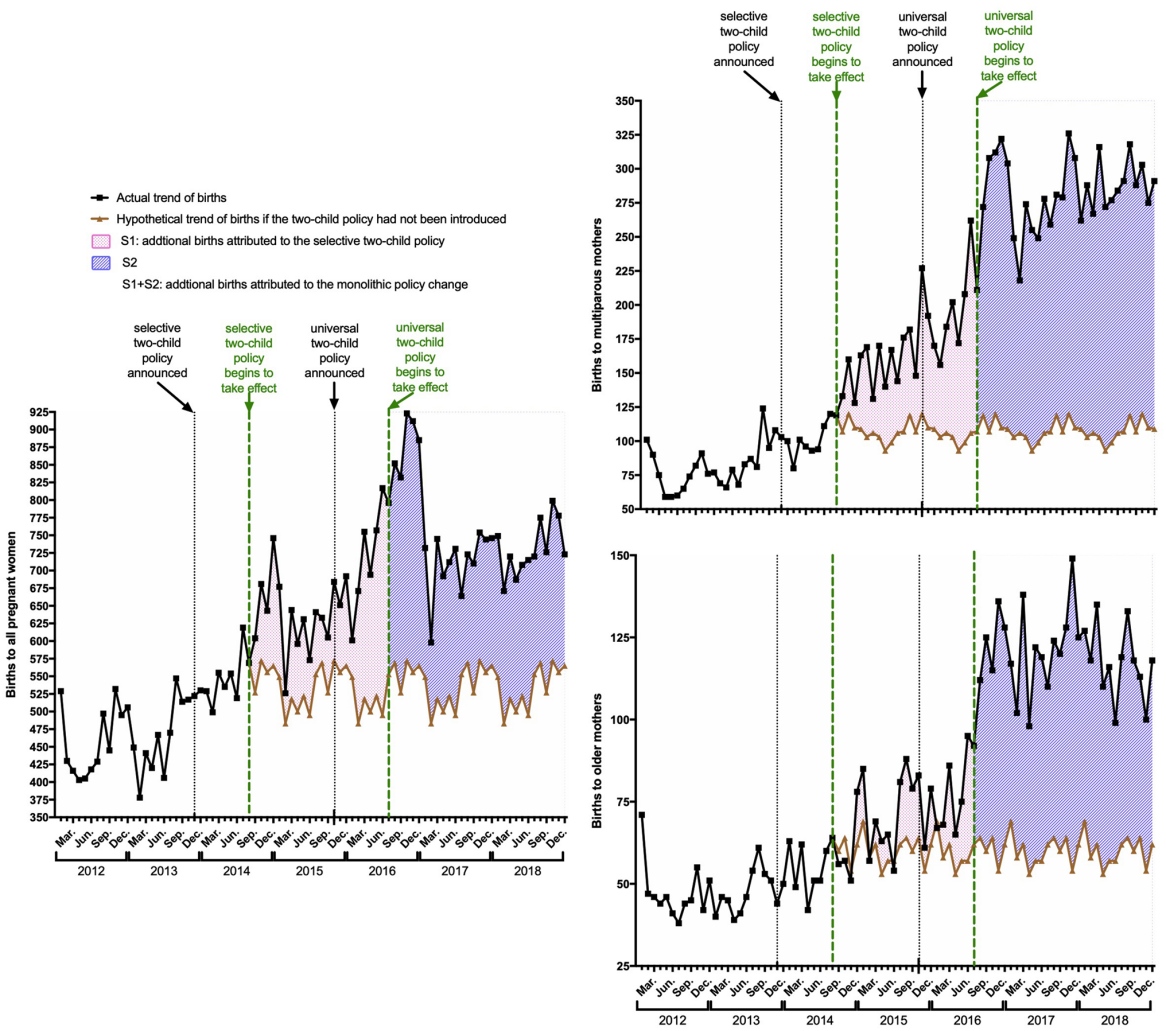
Actual births and hypothetical births. The left panel shows births to all pregnant mothers. The upper right shows births to multiparous mothers, while the lower right shows births to older mothers (≧35 years). The curves of actual births are coloured in black and that of hypothetical births in brown. In each panel, the areas between the two curves could be interpreted as additional births attributed to (1) S1 (pink): the selective two-child policy; and (2) S1 + S2 (pink and purple): the monolithic policy change.

Figure 3 shows the actual and hypothetical curves of births using the approach introduced in the Methods section. The areas could be interpreted as additional births attributed to (1) S1 (pink): the selective two-child policy; and (2) S1 + S2 (pink and purple): the monolithic policy change (both selective and universal two-child policies). The numbers of additional births and the contribution of subgroups are shown in Table 2 simultaneously. Though mothers of advanced age and multiparity are a tiny minority (Table 1), their contribution to additional births is much more obvious (Table 2). Once again, these results lead us to a possible conclusion that older women and multiparous mothers are more susceptible to policy-inspired pregnancy and deserve more attention from care providers and society in the "universal two-child" era.

**Table 2:** The additional births and the contribution of subgroups.

	S1 (23 months)	S2 (30 months)	S1 + S2 (53 months)
Total	2,826	6,362	9,188
Multipara	1,446 (52.2%)	5,201 (81.8%)	6,647 (72.3%)
Advanced age (≧35 years)	244 (8.6%)	1,756 (27.6%)	2,000 (21.8%)

The data could be interpreted as additional births attributed to: (1) S1: the selective two-child policy; (2) S1 + S2: the monolithic policy change (both selective and universal two-child policies).

**Figure 4:**
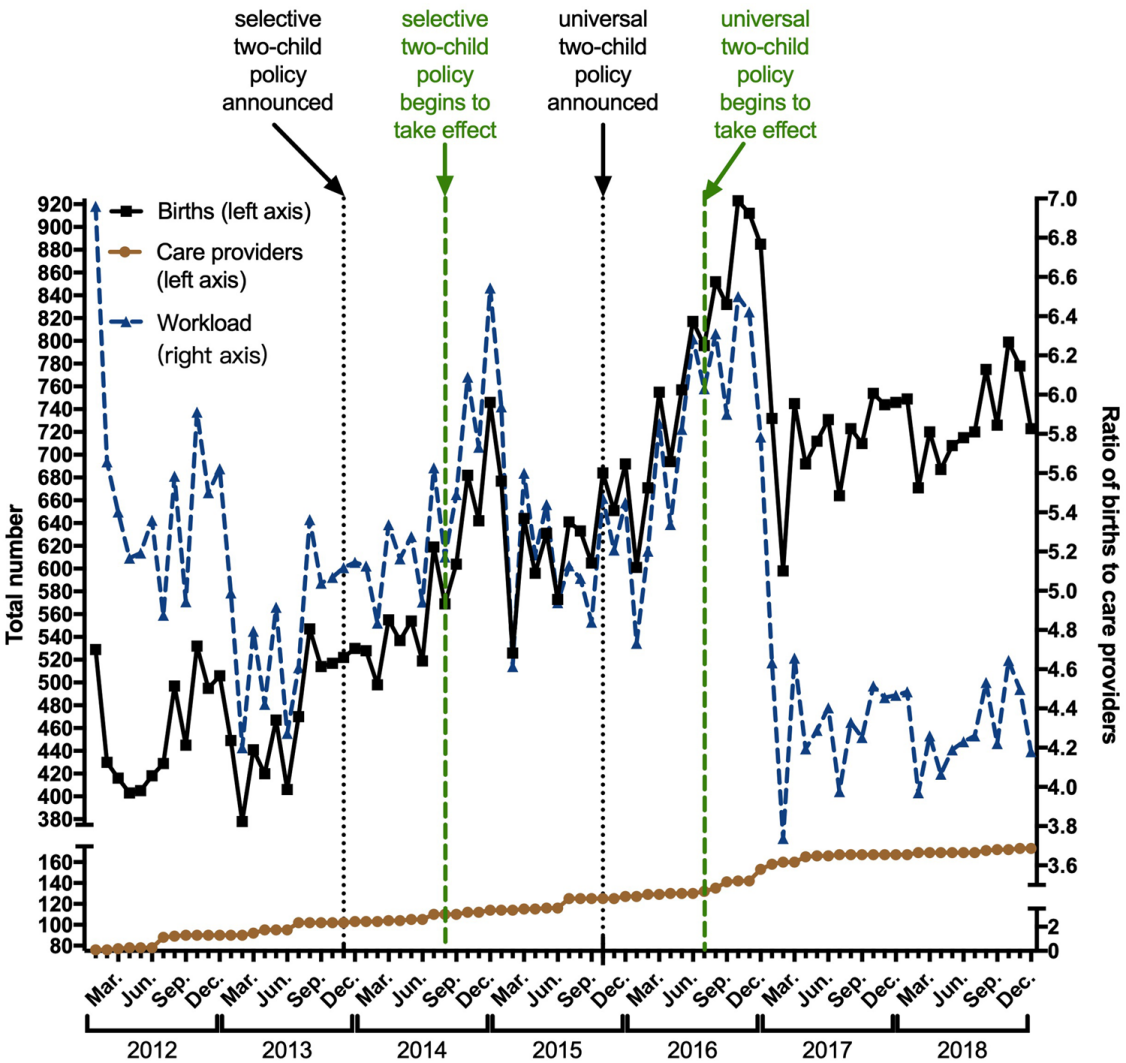
Births and care providers. The temporal patterns of: (1) the absolute number of births (black curve, left axis); (2) the absolute number of care providers (brown curve, left axis); and (3) the workload of care providers, shown by the ratio of births to care providers (blue curve, right axis).

The care providers' workload seems to have decreased after the implementation of the universal two-child policy, with the highest peaks found in the winter of 2016, possibly as a result of the adaptation of recruitment and stabilization of birth (Fig. 4).

### Delivery mode and sex ratio

The CS rate prominently decreased with the shift from the one-child policy to the selective two-child policy and to the universal two-child policy (60.4%, 52.3% and 56.3%, respectively) (Table 3a). Shown in Table 4a, considering age alone, older women were more likely to deliver by caesarean section (odds ratio (OR), 2.36 [2.22, 2.52]), as were multiparous mothers (OR, 1.59 [1.53, 1.66]). When the impacts of age and parity were exempt, the general CS rate has been decreasing over time (OR, 0.68 and 0.73).

**Table 3:** Description of the birth-related obstetrical characteristics.

	Periods			Parity		Age (years)	
	one-child policy	selective two-child policy	universal two-child policy	Nullipara	Multipara	< 35	≧ 35
**a. Maternal**							
CS	60.4	52.3	56.3	52.0	66.9	53.3	76.7
PE	4.3	4.2	4.0	4.0	4.5	3.8	6.8
ICP	2.9	2.4	2.5	2.6	2.5	2.5	3.0
GDM	22.8	17.8	22.6	20.3	23.6	19.3	35.0
PP	5.2	4.7	4.4	3.4	8.2	4.1	9.3
PA	0.5	0.5	0.8	0.6	0.7	0.6	0.8
PTB	4.2	3.4	2.5	3.0	3.9	3.2	3.8
PROM	20.8	23.3	23.2	25.6	15.0	23.4	16.8
Chorioamnionitis	0.2	0.3	1.0	0.6	0.5	0.6	0.5
PPH	0.8	0.9	0.5	0.7	0.7	0.7	0.7
ICU	0.9	0.5	0.5	0.4	1.1	0.5	1.3
Death	NA	0	NA	NA	NA	NA	NA
**b. Neonatal**							
Sex^a^	52.0	52.1	51.8	51.8	52.4	52.0	51.6
Stillbirth	0.6	0.6	0.4	0.5	0.6	0.5	0.6
Deformations	0.2	0.1	0.3	0.2	0.3	0.2	0.3
CA	0.0	0.0	0.1	0.1	0.0	0.0	0.1
FGR	1.7	2.0	2.5	2.1	2.1	2.1	2.4
Macrosomia	5.1	5.3	4.3	4.6	5.4	4.7	5.6
LBW	7.4	6.5	5.2	5.9	6.9	6.0	7.1
Asphyxia	0.1	0.1	0.2	0.2	0.2	0.2	0.1
NICU	0.4	0.3	0.2	0.3	0.2	0.3	0.3

Data are shown in percentage.

NA, not applicable due to its rareness (only one in each group).

*CS* caesarean section, *PE* preeclampsia, a kind of hypertensive disorders in pregnancy, *ICP* intrahepatic cholestasis of pregnancy, *GDM* gestational diabetes mellitus, *PP* placenta previa, *PA* placental abruption, *PTB* preterm birth, *PROM* prelabour rupture of the membranes, *PPH* postpartum haemorrhage, *CA* chromosome abnormality, *FGR* foetal growth restriction, *LBW* low birth weight, *ICU* intensive care unit, *NICU* neonatal intensive care unit.

^a^Presented as percentage of boys.

**Table 4:** Factors influencing the birth-related obstetrical characteristics.

	Periods^a^		Parity^b^	Age (years)^c^
	Selective two-child policy	Universal two-child policy		
**a. Maternal**				
CS	0.68 (0.65–0.72)	0.73 (0.70–0.76)	1.59 (1.53–1.66)	2.36 (2.22–2.52)
PE	0.97 (0.87–1.09)	0.91 (0.81–1.01)	0.91 (0.82–1.02)	1.97 (1.75–2.22)
ICP	0.81 (0.70–0.94)	0.86 (0.75–0.98)	0.90 (0.79–1.03)	1.26 (1.06–1.49)
GDM	0.73 (0.69–0.78)	0.95 (0.90–1.00)	0.93 (0.89–0.98)	2.33 (2.19–2.46)
PP	0.82 (0.74–0.91)	0.66 (0.60–0.73)	2.35 (2.15–2.58)	1.66 (1.50–1.85)
PA	1.02 (0.74–1.40)	1.52 (1.15–1.99)	0.92 (0.71–1.20)	1.31 (0.95–1.80)
PTB	0.78 (0.69–0.88)	0.54 (0.48–0.61)	1.42 (1.27–1.59)	1.07 (0.92–1.24)
PROM	1.22 (1.15–1.29)	1.31 (1.25–1.38)	0.51 (0.48–0.54)	0.89 (0.83–0.96)
Chorioamnionitis	1.57 (0.98–2.51)	5.50 (3.72–8.13)	0.61 (0.46–0.82)	0.89 (0.60–1.33)
PPH	1.21 (0.94–1.56)	0.67 (0.51–0.87)	1.15 (0.90–1.47)	0.98 (0.70–1.37)
ICU	0.57 (0.43–0.75)	0.45 (0.35–0.59)	2.66 (2.08–4.40)	1.64 (1.24–2.15)
Death	NA	NA	NA	NA
**b. Neonatal**				
Sex^d^	1.00 (0.96–1.05)	0.99 (0.95–1.03)	1.03 (0.99–1.08)	0.97 (0.92–1.02)
Stillbirth	1.07 (0.80–1.45)	0.65 (0.48–0.89)	1.50 (1.14–1.97)	0.99 (0.69–1.43)
Deformations	0.62 (0.36–1.07)	1.30 (0.85–1.99)	1.25 (0.83–1.88)	1.07 (0.63–1.82)
CA	1.22 (0.41–3.62)	2.12 (0.84–5.40)	0.38 (0.15–0.97)	4.03 (1.70–9.52)
FGR	1.21 (1.02–1.43)	1.52 (1.31–1.77)	0.90 (0.78–1.04)	1.14 (0.95–1.36)
Macrosomia	1.02 (0.92–1.13)	0.81 (0.73–0.89)	1.21 (1.10–1.33)	1.11 (0.98–1.25)
LBW	0.85 (0.78–0.93)	0.66 (0.60–0.72)	1.25 (1.15–1.36)	1.11 (0.99–1.24)
Asphyxia	1.31 (0.68–2.51)	2.08 (1.17–3.68)	0.83 (0.57–1.56)	0.86 (0.43–1.74)
NICU	0.64 (0.43–0.96)	0.52 (0.35–0.77)	0.86 (0.57–1.30)	1.23 (0.74–2.05)

Data are shown in OR (95% CI). Multivariable logistic regression was used to analyse the impact of policy, age or parity on maternal and neonatal outcomes when controlling for the other two factors.

NA, not applicable due to its rareness (the mortality in some groups are 0).

*CS* caesarean section, *PE* preeclampsia, a kind of hypertensive disorders in pregnancy, *ICP* intrahepatic cholestasis of pregnancy, *GDM* gestational diabetes mellitus, *PP* placenta previa, *PA* placental abruption, *PTB* preterm birth, *PROM* prelabour rupture of the membranes, *PPH* postpartum haemorrhage, *CA* chromosome abnormality, *FGR* foetal growth restriction, *LBW* low birth weight, *ICU* intensive care unit, *NICU* neonatal intensive care unit.

^a^Refer to one-child policy.

^b^Refer to nullipara.

^c^Refer to < 35 years of gestational age.

^d^Calculated by the percentage of boys.

In Table 3b, the neonatal sex ratio, shown as the proportion of new-born boys, is approaching a more balanced status (52.0%, 52.1% and 51.8%), though without statistical significance. However, this was not statistically likely to be attributed to child policy, maternal age, or parity (Table 4b).

### Maternal complications and adverse outcomes

The distribution and influencing factors of maternal complications and adverse outcomes are summarized in Tables 3a and 4a. Older women are more likely to develop PE (preeclampsia) (1.97 [1.75, 2.22]), ICP (intrahepatic cholestasis of pregnancy) (1.26 [1.06, 1.49]), GDM (gestational diabetes mellitus) (2.33 [2.19, 2.46]), PP (placenta previa) (1.66 [1.50, 1.85]) and be referred to the ICU (intensive care unit) (1.64 [1.24, 2.15]). Interestingly, advanced age acts as a protective factor for PROM (prelabour rupture of membranes) (0.89 [0.83–0.96]). In this study, multiparity is protecting mothers from GDM, PROM and chorioamnionitis but is a risk factor in PP, PTB (preterm birth) and ICU admittance. Furthermore, not considering the two covariates, policy changes alone have probably decreased the rates of some adverse outcomes, including ICP, GDM, PP, PTB, PPH (postpartum haemorrhage) and ICU admittance, but increased those of PA (placental abruption) and chorioamnionitis, especially when conducting comparisons between the periods of the universal two-child policy and the one-child policy.

### Neonatal complications and adverse outcomes

The outcomes, illustrated in Tables 3b and 4b, remind us of the low incidence and high importance of neonatal adverse events. Pregnant woman aged 35 years or older have a much greater risk (approximately 4.03 times) of having a baby with a chromosomal abnormality. When compared with nullipara, multipara is associated with an increased likelihood of stillbirth or having a baby that suffers from macrosomia or LBW (low birth weight), but these babies are less likely to carry abnormal chromosomes. The positive effects of policy changes can be seen in many of the outcomes (stillbirth, macrosomia, LBW and neonatal intensive care unit admittance), while negative influences are found only in FGR (foetal growth restriction) and related asphyxia.

## Discussion

In this study, data from over 50,000 individuals were analysed, which is quite sufficient for explaining scientific questions from statistical points of view. The probability of bias generated from selective or false recording is low because of the strict standards for the medical recording process in our hospital, which requires junior doctors to document promptly and seniors to check documentation carefully for every single patient. However, though extremely rare (3.49%), some incomplete data do exist, which might be a source of attrition bias. Most importantly, this is a single-centred study, and the representativeness of samples directly affects the applicability of our findings. Considering that our hospital is a large tertiary care centre in southwest urban China, the current results are appropriate for use in southwest regions centred on Chongqing, but their accuracy and possible variation should be taken into consideration in regard to other areas. A detailed discussion on the principal findings and the specific strengths and limitations is presented below.

### Trend of deliveries and change of background characteristics among pregnant women

The pattern of deliveries in our study (Fig. 2a) was similar to the national pattern^7^. The trend of deliveries peaked twice, in the winters following each policy announcement, and finally became stable after 2016.

We believe that deliveries reached a plateau after 2016 because of the saturation of the policy's target. In other words, the maximum fertility potential of target women was quickly realised during this short period, reaching a greater balance with a new background distribution of mothers. To demonstrate this newly balanced distribution, we found that the proportion of older and multiparous mothers increased (Table 1), which was also noted by Li and colleagues^7^. The policy change has only been in effect for 3 years, and more nationwide and longer-term data are needed to guide reproductive strategy in the future.

Notably, the changes in frequency of abortion or miscarriage, which we show in Fig. 2a, deserve to be studied more deeply.

### Additional births

The aim of the universal two-child policy was to increase the annual births by one million to more than 10 million^4,12^. Although our data are far from comparable to national data, the additional births in one hospital are quite considerable (Fig. 3 and Table 2), at approximately 123 and 173 per month, considering the change from the one-child policy to the selective two-child policy and the monolithic policy change, respectively. Noteworthily, the model of hypothetical births might result in bias.

### The target of the reproductive policy

To identify the target of the reproductive policy, the actual change in the background population structure (the increase in proportion) was estimated (Table 1). In addition, we considered that contribution of additional births among subgroups to the general group (the proportion of increment) could serve as additional evidence (Table 2). Of note, the validity of our model on hypothetical births might bias our results almost equally to each group, but this would not change to an extent that reverses our conclusion. This procedure, from modelling to obtaining contribution and to interpreting, is one of the highlights of our study and is suitable for guiding other similar studies.

According to our study, all women would be potential targets of a reproductive policy, though multiparous mothers and those of advanced age were more likely than others to respond. In contrast, another researcher defined in advance that nulliparous births would not be affected by the policy^7^. Surprisingly, our conclusion is the same as his, that the increase in births was largely driven by those to mothers who had already delivered one baby^7^.

### The condition of medical care

We creatively found that, though the workload of care providers has not increased, in sight of the number of births (Fig. 4), the excessive increase of high-risk births (Tables 1, 3) makes the adaptation of health-care resources inappreciable. Moreover, the number of births handled by one medical staff, which is at least 4 according to our study, is far from rational. Similar concerns about health systems exist nationwide, not only for the work quantity, but also quality^13–15^. The rapid adaption of the medical system to the soaring needs and expectations demands more efforts from all of us.

### Delivery mode and sex ratio

First, after the announcement of the two-child policy, the CS rate declined steadily in all regions of China^16,17^. In our study, the rate of CS decreased but was still high (more than one half). Second, advanced age was a strong risk factor for CS (OR, 2.36). Similar to the case in our previous study^9^, nulliparous women experienced a lower incidence of CS (OR, 1.59), suggesting a preference for vaginal birth in the first delivery, while multiparous mothers were more likely to have second caesarean sections because of the concerns about the safety of VBAC (vaginal birth after caesarean section)^7^. Third, in the one-child policy era, the public belief that caesareans were relatively safer promoted CS to some extent^18^. Reassuringly, as long as age and parity permitted, the pregnant women in hospital were becoming less willing (approximately 0.7 times) to choose CS during our study period, suggesting the effectiveness of various Chinese guidelines intended to reduce the CS rate and pursue baby-friendly deliveries^19^. Lastly, this study was conducted in southwest urban China. The CS rate in rural areas was lower; therefore, our numbers could represent an overestimate. To sum up, more work is needed to control the CS rate and to ensure the health of the maternal population, particularly women with advanced age and multiparity.

One of the worst impacts of the one-child policy is the skewed sex ratio at birth^20–22^. In our study, the sex bias decreased, which is good news. This process of sex-ratio balance was also demonstrated around 2007^23^ and it was developed with progress in gender equality and women's empowerment in China.

### Complications and adverse outcomes

#### Association with age

Some previous studies from other regions found a controversial frequency of maternal complications and adverse outcomes in the context of the universal two-child policy^24–27^. However, in our study, the actual percentages of those maternal adverse outcomes decreased with the policy changes, except for GDM (Table 3a). We would like to emphasize that there were many more older mothers in the periods of the universal two-child policy (Table 1), and the negative influences of age were found not only by us (Table 4a), but by various studies^28–30^. It is not difficult to imagine that those studies conducted comparisons without consideration of some covariates, such as age, and the influence of policy they identified was the general effect of all factors, which is hard to interpret as one unified conclusion.

Though a series of adverse neonatal outcomes, such as low birth weight, macrosomia and chromosome abnormality, are considered to be relevant to advanced maternal age^10,30^, we only found its association with chromosome abnormality (Table 4b). The decrease in genetic stability over time, resulting in a greater potential for chromosome abnormality in the baby, might be the explanation for this finding, which undoubtedly reminds us that the specific categories of CA and their potential to be detected earlier deserve more attention and research in the new era.

**Figure 5:**
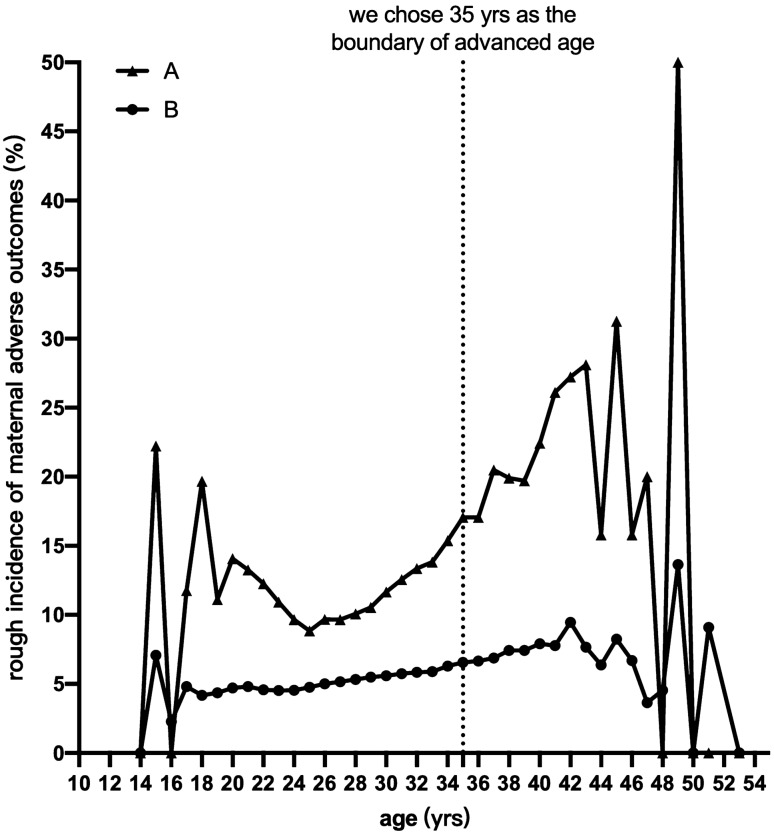
The association between maternal age and adverse outcomes. In the line chart, the X axis indicates the maternal age, while the Y axis indicates the incidence of maternal adverse outcomes, shown as a percentage. All maternal adverse outcomes were equally weighted, including PE, ICP, GDM, PP, PA, PTB, PROM, chorioamnionitis, PPH, ICU admittance, and death. Curve A: the numerator is the number of mothers who experienced at least one of the 11 outcomes, and the denominator is the total number of mothers at the corresponding age. Curve B: cumulative outcomes, meaning that the numerator is the total frequency of all 11 outcomes, while the denominator is 11 times the number of mothers.

In addition, though it is widely acknowledged that advanced maternal age is a risk factor for adverse maternal and neonatal outcomes, a clearly age-driven increase in those events and an exact cut-off age for increased risk are difficult to determine because of the complexity of covariates and the numerous types of adverse events. A previous study found a significantly higher frequency of GDM and PE in very-advanced-aged women (45–59 years)^29^, and another conducted similar analyses on three age groups (20–34, 35–39, and ≧ 40 years)^28^. In our study, we chose 35 years as the boundary of advanced age somewhat arbitrarily, based on our rough portrayal of the age-event curve (Fig. 5). The selection of cut-off age, however, would undoubtedly affect the conclusion. Our strategy for this issue, shown in Fig. 5, can only be feasible at an individual level, and there are two problems to be considered: control of covariates and assignment of weight to various outcomes. Our research group plans to solve these scientific questions by machine learning in the future.

#### Association with parity

In these 7 years of rich data, we found that parity might act as both a protective factor and a risk factor in adverse maternal and neonatal outcomes. A study concerning whether the so-called "dangerous multipara" is a factor or just fiction finally concluded that adverse outcomes were not related to parity^17^. Nevertheless, the most remarkable result concerning parity's effects in this study demonstrates that multiparous women were 2.35 times as likely to have placenta previa and 2.66 times as likely to be admitted to the ICU than nullipara. Given that multiparous women probably experienced CS in their previous delivery, which is an independent risk factor for PP and ICU admittance^17^, this higher risk is understandable. Fortunately, for instance, nulliparous women prefer vaginal deliveries to CS if they ultimately want to have two babies, and thus the rates of PP and ICU admittance among multipara might decrease in the long run, despite the increase in the current study. We are unsure about the decreased likelihood of CA with increasing parity, probably as a result of a filtering function of previous delivery for the multipara.

#### Association with policy alone

Regardless of covariates such as age and parity, the effect of policy alone was generated from the complex of many social factors, including time-dependent macroscopic SES (socioeconomic status), overall medical condition, and public sentiment. In terms of this study, many adverse pregnancy outcomes decreased over time (Table 4), reflecting the extraordinary progress in medical technology and management. Nevertheless, it is worth noting that the incidence rates of PROM and chorioamnionitis are on the rise. This issue deserves more attention to determine whether these increases originated from irregular or unnecessary operations, and it suggests us to adjust clinical practice once the problems are identified. Nevertheless, the individual SES level, which has not been controlled for in our regression model, could act as a potential confounding factor of the policy's effect. To some extent, the association measures observed may be overestimated, especially for abortion, caesarean delivery, preeclampsia, preterm delivery, and obstetrical haemorrhage, which have been demonstrated to be correlated to SES status^31^. Similar bias should also be considered when interpreting our findings.

This kind of analysis process to identify the effects of time, regardless of its covariates, is researcher-friendly. Generally, further research will be needed to develop a more nuanced understanding of the sustained impact of this historical change in China.

### Highlights

To our knowledge, this is a novel study in the obstetrics field, which can serve as a paradigm and provide both clear and practical analytical processes for investigating the influences of time series and multi-covariates, particularly when comprehensive national-level data are hard to acquire. Such procedures possess the advantage of showing more details over the macroscopic descriptions of big data.

## Conclusions

Our study, conducted in a representative hospital in southwest China, demonstrated that (1) an increase in births occurred after implementation of the two-child policy and has already stabilized; (2) a newly balanced demographic makeup of pregnant women has developed; (3) the policy target is more sensitive to additional births; (4) the policy put hospitals and care providers in a situation of lower quantity but greater difficulty work; and (5) the covariates should not be ignored when analysing the factors influencing the birth-related obstetrical characteristics.

We sincerely recommend that (1) more individual-based studies be performed both to describe changes and to answer the questions mentioned above; (2) more efforts should be put into providing high-quality health care service to prevent adverse outcomes from improper operations; and (3) childbearing should be concentrated within the optimum maternal age range.

## Methods

### Study design and data collection

This retrospective observational study was conducted in the First Affiliated Hospital of Chongqing Medical University, an institution-based tertiary care centre in southwest urban China. Individual delivery information was obtained for all pregnant women referred to the Department of Obstetrics from January 2012 to December 2018, and all available records were considered eligible. The records included those related to (1) identity: interpatient number; (2) time series: date of delivery; (3) maternal background: age, parity, foetal number, and method of conception; (4) delivery mode and neonatal sex; (5) maternal complications and adverse outcomes; (6) neonatal complications and adverse outcomes. We excluded those (1) with important missing information that could not be supplied manually; and (2) who delivered before 28 weeks of gestation (defined as abortion or miscarriage in this study). Of note, data regarding care providers were obtained from the HR (Human Resources) department of our hospital.

### Ethics approval

This study was conducted ethically in accordance with the World Medical Association Declaration of Helsinki. The institutional ethics committee of the First Affiliated Hospital of Chongqing Medical University reviewed and approved this study (20172601), and the study was exempt from the requirement for informed consent.

### Definition

Family planning policies were considered to take effect nine months and five months after the announcement for deliveries or births and for abortions or miscarriages, respectively. Therefore, the time series for deliveries or births were divided into 3 periods: (1) one-child policy: from January 1st, 2012 to July 31st, 2014; (2) selective two-child policy: from August 1st, 2014 to June 30th, 2016; and (3) universal two-child policy: from July 1st, 2016 to the end of 2018.

Care providers were defined as the total of obstetricians, paediatricians, sonographers and nurses.

Preterm birth was defined, according to Chinese guidelines, as delivery occurring between 28^+0^ and 36^+6^ weeks of gestation. Similarly, those deliveries that occurred before 28 completed weeks of gestation were defined as abortions or miscarriages. All other complications and adverse pregnancy outcomes were defined following international obstetrical practice.

### Analysis

#### Displaying temporal patterns

Monthly absolute values or percentages of factors are visually shown in the figures (Prism 8 for Windows, Version 8.0.1 (244); GraphPad Software Inc, San Diego, CA).

#### Exploring the change of background characteristics among pregnant women

In the time series, comprised of 3 periods, the background variations of pregnant women were estimated using chi-squared tests (IBM SPSS Statistics 20.0) based on the constituent ratio of the demographic and pre-delivery characteristics: age group, parity, number of foetuses, and method of conception. The chi-squared tests were two-sided and were regarded as statistically significant when the p-value was lower than 0.05.

The statistical significance and its clinical significance were considered as the eligibility criteria for choosing potential subgroups and defining covariates in subsequent analyses.

#### Estimating additional births and care providers' workload.

Through referring to the annals of statistics for China^32^, we can assume that the area covered by the service of our hospital and the number of women of childbearing age in this area remained consistent during the study period, and their composition was stable during the period of the one-child policy. Thus, the monthly mean number of births (subject to neonatal numbers) would have continued to trend in parallel if new policies had not been introduced. To mimic the birth curves free of policy impacts, we used the average number of births in corresponding months to describe the annual pattern and created a translation of this curve to a start point at the end point of the former one-child period. As a result, an actual curve and a hypothetical curve were generated for the general group and subgroups (chosen in the step of "background characteristics"), respectively. The area between the actual curve and the hypothetical curve could be interpreted as the theoretical additional births attributed to the shift in policy. The rate of additional births in a subgroup to the general group could be considered as the policy-response contribution of the corresponding subgroup factor.

The care providers' workload in each month, calculated by dividing the number of births by the number of care providers, could serve as a reference for evaluating the conditions of medical care.

#### Identifying maternal and neonatal outcomes

The birth-related obstetrical characteristics, including delivery mode, neonatal sex ratio, complications and adverse outcomes, were described as proportions in various groups. A multivariable logistic regression was conducted (IBM SPSS Statistics 20.0) to assess the factors influencing these obstetrical outcomes. The policies and the covariates (chosen in the step of "background characteristics") were included in the regression model, and the impact of each variate was determined when controlling for the others. P values < 0.05 were considered statistically significant.

## Data Availability

The data used and analysed during the current study are available from the corresponding author on reasonable request.

## Abbreviations

ART: Assisted reproductive technology
IVF-ET: In vitro fertilization and embryo transfer
HR: Human resources
CS: Caesarean section
PE: Preeclampsia, a kind of hypertensive disorders in pregnancy
ICP: Intrahepatic cholestasis of pregnancy
GDM: Gestational diabetes mellitus
PP: Placenta previa
PA: Placental abruption
PTB: Preterm birth
PROM: Prelabour rupture of the membranes
PPH: Postpartum haemorrhage
CA: Chromosome abnormality
FGR: Foetal growth restriction
LBW: Low birth weight
ICU: Intensive care unit
NICU: Neonatal intensive care unit
VBAC: Vaginal birth after caesarean section
OR: Odds ratio
SES: Socioeconomic status

## References

[1] The end of the one-child policy in China? *Lancet (London, England)* **377**, 968 (2011). 10.1016/S0140-6736(11)60369-3 21420540

[2] Hesketh T, Lu L, Xing ZW (2005). The effect of China's one-child family policy after 25 years. N. Engl. J. Med..

[3] Hesketh T, Zhou X, Wang Y (2015). The end of the one-child policy: lasting implications for China. JAMA.

[4] Zhai ZW, Li L, Chen JJ (2016). Accumulated couples and extra births under the universal two-child policy. Popul. Res..

[5] The two-child policy in China: what to expect? *Lancet (London, England)* **382**, 1758 (2013). 10.1016/S0140-6736(13)62534-9 24290582

[6] Zeng Y, Hesketh T (2016). The effects of China's universal two-child policy. Lancet (London, England).

[7] Li HT, et al. Association of China's universal two child policy with changes in births and birth related health factors: national, descriptive comparative study. *BMJ (Clin. Res. ed.)* **366**, l4680. 10.1136/bmj.l4680 (2019). 10.1136/bmj.l4680 PMC6699592 31434652

[8] Dietl A, Farthmann J (2015). Gestational hypertension and advanced maternal age. Lancet (London, England).

[9] Zhao J, et al. Effect of second child intent on delivery mode after Chinese two child policy implementation: a cross sectional and prospective observational study of nulliparous women in Chongqing. *BMJ Open* **7**, e018823. 10.1136/bmjopen-2017-018823 (2017). 10.1136/bmjopen-2017-018823 PMC5770909 29282269

[10] Cleary-Goldman J (2005). Impact of maternal age on obstetric outcome. Obstet. Gynecol..

[11] Salem Yaniv S (2011). A significant linear association exists between advanced maternal age and adverse perinatal outcome. Arch. Gynecol. Obstet..

[12] Meng LG, Bo LI, Chen L. Study on the influence of “full two-child”policy on incremental population and aging population. *J. Guangdong Univ. Financ. Econ.* (2016) (**(in Chinese)**).

[13] Hu KJ, Sun ZZ, Rui YJ, Mi JY, Ren MX. Shortage of paediatricians in China. *Lancet (London, England)* **383**, 954 (2014). 10.1016/S0140-6736(14)60482-7 24629297

[14] Ren Z, Song P, Theodoratou E, Guo S, An L (2015). China’s human resources for maternal and child health: a national sampling survey. BMC Health Serv. Res..

[15] Song Q, Wang F, Zhuang N. The pediatric demands and gaps under the universal two child policy. *Chin J Health Policy*, 65–70 (2016).

[16] Feng XL, Wang Y, An L, Ronsmans C (2014). Cesarean section in the People's Republic of China: current perspectives. Int. J. Women's Health.

[17] Wang E, Hesketh T (2017). Large reductions in cesarean delivery rates in China: a qualitative study on delivery decision-making in the era of the two-child policy. BMC Pregnancy Childbirth.

[18] Liang J, et al. Relaxation of the one child policy and trends in caesarean section rates and birth outcomes in China between 2012 and 2016: observational study of nearly seven million health facility births. *BMJ (Clin. Res.)* **360**, k817. 10.1136/bmj.k817 (2018). 10.1136/bmj.k817 PMC5836714 29506980

[19] National Health and Family Planning Commission. The name list of baby friendly hospitals in China. https://www.nhc.gov.cn/fys/s7906/201511/e5650712dbcd449e9d2e01129a698b9c.shtml (2015).

[20] Yi Z (2007). Options for fertility policy transition in China. Popul. Dev. Rev..

[21] Yang J (2006). Regional diversity of fertility and child sex ratio in China. Popul. Res..

[22] Song Y, Chen R. A micro empirical study of the effect of family planning policy on sex ratio at birth. *Popul. Res*. 44–49 (2009) (**(in Chinese)**).

[23] Guo Z. Multi-level analysis on the sex ratio at birth in China based on the 2000 census and the regional fertility policy data. *Popul. Res*. 20–31 (2007) (**(in Chinese)**).

[24] Cakmak Celik F, Aygun C, Kucukoduk S, Bek Y (2017). Maternal and neonatal outcomes in advanced maternal age: a retrospective cohort study. J. Mater. Fetal Neonatal Med..

[25] Shan D (2018). Pregnancy outcomes in women of advanced maternal age: a retrospective cohort study from China. Sci. Rep..

[26] Zhang HX, Zhao YY, Wang YQ (2018). Analysis of the characteristics of pregnancy and delivery before and after implementation of the two-child policy. Chin. Med. J..

[27] Liu Y (2019). Changes of second-time mothers and their infants under the universal two-child policy in Changsha China. Midwifery.

[28] Arya S, Mulla ZD, Plavsic SK (2018). Outcomes of women delivering at very advanced maternal age. J. Women's Health.

[29] Laskov I (2012). Outcome of singleton pregnancy in women ≥ 45 years old: a retrospective cohort study. J. Mater. Fetal Neonatal Med..

[30] Frederiksen LE (2018). Risk of adverse pregnancy outcomes at advanced maternal age. Obstet. Gynecol..

[31] Kim MK (2018). Socioeconomic status can affect pregnancy outcomes and complications, even with a universal healthcare system. Int. J. Equity Health.

[32] National Bureau of Statistics. CHINA STATISTICAL YEARBOOK. https://www.stats.gov.cn/tjsj/ndsj/ (2019).

